# Effect of acid hydrolysis on the structural and antioxidant characteristics of β-glucan extracted from Qingke (Tibetan hulless barley)

**DOI:** 10.3389/fnut.2022.1052901

**Published:** 2022-11-10

**Authors:** Lan Zhao, Shuwei Lin, Jingying Lin, Jia Wu, Huibin Chen

**Affiliations:** ^1^School of Food Science and Engineering, College of Life Sciences, Fujian Normal University, Fuzhou, China; ^2^College of Biological Science and Engineering, Fuzhou University, Fuzhou, China

**Keywords:** Qingke β-glucan, phytochemicals, acid hydrolysis, structural characterization, antioxidant activity

## Abstract

In this study, we explored the effect of acid hydrolysis on the molecular, structural, rheological, thermal, and antioxidant characteristics of Qingke β-glucan. The acid hydrolysis reduced the molecular weights of β-glucans from 510 to 155 KDa. The results of the structural analysis by nuclear magnetic resonance (NMR) spectroscopy, X-ray diffraction, and fourier transforms infrared (FTIR) spectroscopy indicated that acid hydrolysis did not change the primary functional groups of β-glucans. The rheological behavior of β-glucan without and with acid hydrolysis can be described as pseudoplastic and Newtonian, respectively. The DSC curves of the β-glucans with high molecular weights showed the highest transition temperature. The 2, 2’-azino-bis(3-ethylbenzothiazoline-6-sulfonic acid) (ABTS) radical cation scavenging activity and the reducing power of soluble β-glucans in Qingke showed a dose-dependent pattern. Meanwhile, the antioxidant activities of Qingke β-glucan of different molecular weights were similar. This study demostrated that the acid hydrolysis almost have no effect on antioxidant activity of Qingke β-glucans.

## Introduction

β-glucan is a homopolysaccharide of D-glucopyranose linked by (1→3) and (1→4) glycosidic linkages in cereals and (1→3) and/or (1→6) glycosidic linkages in fungal sources, respectively (1). Various β-glucans have been isolated from sources like fungus, barley, oats, and seaweed. Depending on the aspects of their main structure, such as the type of linkage, degree of branching, molecular weight, and conformation (2), β-glucans have different physicochemical properties. β-glucans of Qingke can prevent and manage type 2 diabetes (3), lower cholesterol (4), improve inflammation (5), and mediate metabolic disorders (6).

The primary food of the Tibetan people and an essential component of the diet of livestock in the Tibetan plateau is Qingke. Qingke is a type of hulless barley that is found at high altitudes (7). It is a crucial crop for global production, but the food industry only uses it as feed and malt (8). Qingke has attracted attention as a functional food past 10 years owing to its health benefits, mainly because Qingke flour is rich in soluble dietary fibers, particularly β-glucan. β-glucans are remarkable quality markers for the assessment of Qingke cultivars. β-glucan has potent antioxidant activities (9). The characteristics of β-glucan are significantly influenced by its molecular structure and several studies have shown that the antioxidant activities of the Qingke β-glucan can be effected by molecular structure or weight (10). In experimental animals model of lipopolysaccharide-induced chronic enteritis (11), oat β-glucan of high molecular weight is more effective to reduce stress oxidation. Moreover, Shah et al. (12) reported that gamma-irradiated barley produced low molecular weight β-glucan with high antioxidant activity. The degradation of the molecule can be facilitated by various chemical and physical processes, including thermal treatment, oxidative stress, enzymatic hydrolysis, and radiation (2). Meanwhile, Jia Wu et al. (13) reported that acid hydrolysis could reduce the molecular weights of oat β-glucan. In the previous study, the relationships between the different molecular weights of Qingke β-glucans by acid hydrolysis and binding properties *in vitro*, inhibition of digestive enzymes, anti-inflammatory activities, and anti-cancer activities were extensively investigated (7).

However, it is yet unclear if the acid hydrolysis had an impact on the physicochemical and antioxidant properties of Qingke β-glucan. In this research, we aim to compare the effects of acid hydrolysis on the molecular, structural, and antioxidant capacities of Qingke β-glucan. The β-glucan of different molecular weights by acid hydrolysis was obtained and further examined by molecular weight determination, methylation analysis, nuclear magnetic resonance (NMR) spectroscopy, fourier transforms infrared (FTIR) spectrometry, X-ray diffraction (XRD) measurement, thermal properties measurement, and apparent viscosity measurements. Additionally, the antioxidant capabilities of Qingke β-glucans were assessed *in vitro*. The results of this research indicated the β-glucan from Qingke as a novel antioxidant in the pharmaceutical and functional food-related industries and provided fundamental information about the composition and biological characteristics of Qingke β-glucans.

## Materials and methods

### Material and chemical reagents

Qingke was procured from regional research station, Tibet, China. The ABTS, dichloromethane, trichloroacetic acid, and iodomethane was bought from Shanghai Sigma-Aldrich Biotechnology Co. Other reagents were bought from Sinopharm Chemical Reagent Co., Ltd.

### Preparation and purification of Qingke β-glucan

β-glucan was isolated from Qingke flour by the double-enzymatic method as reported by Lazaridou et al. (14), with some adjustments. The extraction process is as follows:

Qingke powder was refluxed with 82% ethanol for 3 h at 85°C (1:5, w/v). After centrifugation (4,000 rpm) for 10 min, ethanol (82%) washes were conducted on the pellet twice. After centrifugation again (4,000 rpm) for 10 min, the precipitate was kept and dried at 40°C for 24 h. Extraction of β-glucan was performed twice at 52°C in a water bath for 2 h (1:10, w/v). Then, centrifugation (4,000 rpm) was conducted for 30 min. The supernatant was concentrated to one-third by rotary evaporation at 60°C. Calcium chloride and thermostable α-amylase enzyme were added at 80°C for 30 min. The pH was adjusted to 8.0, and pancreatin digestion was performed at 38°C for 3 h. After centrifugation (5,000 rpm) for 30 min, the supernatant was obtained, the residue was removed, and the pH was adjusted to 7.0. A certain volume of 95% ethanol solution was added for 12 h twice and was centrifuged (5,000 rpm) for 30 min, and the precipitate was redissolved in distilled water at 85°C. The supernatant was further processed by ultrafiltration and centrifugation (molecular weight cut off: 5 kDa) and the sample was vacuum freeze-dried. Finally, purified Qingke β-glucan was obtained.

### Partial hydrolysis with acid

Qingke β-glucans of low molecular weights were obtained by acid hydrolysis following the method described by Wu et al. (13). For preparing 1% original Qingke β-glucan (QBG) solutions, a certain amount of HCl was added, and the final mass fraction of HCl was 0.1 mol/L. The prepared solution was then stirred magnetically in a water bath at 80°C for 30 min (QBG30), 60 min (QBG60), and 90 min (QBG90). Then, the hydrolysate was immediately cooled to 25°C and neutralized with NaOH solution. A double volume of absolute ethanol was applied to precipitate the partially hydrolyzed Qingke β-glucan. Then, the precipitates were redissolved in water and was further processed by ultrafiltration and centrifugation (molecular weight cut off: 5 kDa). Finally, the sample was vacuum freeze-dried.

### Molecular weight determination

Following the method described by Yang et al. (15), the molecular weight of Qingke β-glucan was measured using SEC-MALLS-RI. By a DAWN HELEOS-II laser photometer (Wyatt, USA) equipped with an SB-803 HQ and an SB-804 HQ column (Showa Denko, Japan), the molecular weight of Qingkes β-glucans was measured in a 0.1 mol/L NaNO_3_ aqueous solution, including 0.03% NaNO_3_, with a flow rate of 0.45 ml/min. The temperature was monitored at 45°C by a model column heater (Sanshu Biotech, Shanghai, China). The concentration of the Qingke β-glucans is 1 mg/ml. A differential refractive index detector (Wyatt, USA) was used to detect the dn/dc value. The refractive index increment (dn/dc) value of the fractions was set to be 0.142 ml/g. Chromatographic data was processed with software ASTRA 6.1.

### Methylation analysis

The Qingke β-glucan samples were methylated using iodomethane following the method reported by Wu et al. (13). The methylated β-glucans were hydrolyzed with 100 μL 2 mol/L trifluoroacetic acid for 90 min at 121°C in a fan-forced oven, then add 50 μL 2 mol/L ammonium hydroxide and 50 μL 1 mol/L sodium borodeuteride to react at room temperature for 2.5 h and then were acetylated with 250 μL acetic anhydrid at 110°C for 2 h. Then, the product was dissolved in 500 μL dichloromethane, followed with linkage analysis by a gas chromatography-mass spectrometer (GC-MS) (7890B/5977A, Agilent, USA) connected with an HP-5MS capillary column (30 m × 0.25 mm × 0.25 μm). Mass spectrometry analysis was preheated to a starting temperature of 150°C, held for 5 min, accelerated to 280°C at a speed of 6°C/min, then maintained for 5 min. Therefore, this experiment mainly calculates the ratio of the chromatographic peak area to the molecular weight of the corresponding derivative by GC-MS, and then calculated the relative molar ratio of various glycosidic linkages in the Qingke β-glucans.

### Nuclear magnetic resonance spectroscopy

Nuclear magnetic resonance spectroscopy was recorded using the JEOL-ECZ600R NMR spectrometer (JEOL Japan, Japan). In total, 12 mg of β-glucan was dispersed in 0.6 ml dimethyl sulfoxide (DMSO-*d*6) for ^1^H spectra and ^1^H-^1^H correlation spectroscopy (COSY) at 80°C. Meanwhile, same samples was dispersed in 0.6 ml D_2_O for ^13^C NMR spectroscopy and ^1^H–^13^C heteronuclear single-quantum coherence spectroscopy (HSQC) at 25°C.

### Fourier transform infrared spectroscopy

Under an infrared lamp, 2 g of KBr and a moderate amount of β-glucan samples were mixed and squeezed into a rounded tablet. The FT-IR spectroscopy was performed using a Nicolet IS50 FT-IR spectrometer (Thermo Scientific, USA) with a 400–4,000 cm^–1^ wavenumber range.

### X-ray diffraction measurement

Using an X-ray diffractometer (Empyrean, Netherlands) equipped with Cu-Kα radiation, the Qingke β-glucan samples were scanned in the range of 4° < 2θ < 50° with a scanning speed of 0.5°/s and scanning 10 s per step. The technique was operated at a tube pressure and tube flow of 40 kV and 40 mA, respectively.

### Measurement of thermal properties

Thermal gravimetric analysis (TGA) and differential scanning calorimetry (DSC) tests were conducted using a simultaneous thermal analyzer (Netzsch, STA449F5, Germany). The pans were sprayed with 5.5 mg of β-glucan, which was heated from 30 to 400°C at a speed of 10°C/min. Indium was used to calibrate the instrument, and an empty pan served as the reference.

### Apparent viscosity measurement

Samples of Qingke β-glucans were dissolved in distilled water at a 2% concentration (w/w). The apparent viscosity of Qingke β-glucan solutions at 25°C was tested by a Physica MCR-302 rheometer (Anton Paar, Austria) with a 50 mm diameter cone plate (CP-60) according to a described method (13). There was an increase in shear rate from 0.1 to 1000 s^–1^.

### Antioxidant activity *in vitro*

#### ABTS radical cation scavenging activity

We used a method reported by Bai et al. (16) with slight modifications, to explore the ABTS radical scavenging activity of Qingke β-glucans. The ABTS^+^ stock solution was prepared by combining 7.4 mmol/L ABTS solution (10 ml) with 2.5 mmol/L potassium persulfate (10 ml) and leaving it in the dark for 16 h at room temperature. When required, the phosphate-buffered saline (PBS)-diluted ABTS^+^ radical cation solution had the absorbance of 0.75 at 734 nm. Various concentrations (from 4, 6, and 8 mg/ml) of β-glucan solution (0.2 ml) were mixed with the prepared ABTS^+^ radical cation solution (2 ml) for 1 h in the dark. The mixture was centrifuged at 3,000 rpm for 10 min to remove the residue. The absorbance of the supernatants was measured at 734 nm. The ABTS radical cation scavenging activity was evaluated using the following equation:


$$\mathrm{Effect}\left(\%\right)=\frac{\mathrm{A1}-\mathrm{A2}}{\mathrm{A1}}\times 100\%$$

Here, A_1_ is the absorbance of the mixture of water and ABTS^+^ radical cation solution and A_2_ is the absorbance of the mixture of β-glucan and ABTS^+^ radical cation solution.

#### Reducing power

The reducing power of the Qingke β-glucan was reported as described by Zhu et al. (17) with few modifications. Each sample (QBG, QBG30, QBG60, and QBG90) of different concentrations (4, 6, and 8 mg/ml) was mixed with 0.25 ml potassium ferricyanide (1 g/100 ml), and 0.5 ml of the phosphate buffer (0.2 mol/L, pH = 6.6). The mixture was incubated at 50°C for 30 min and centrifuged for 10 min at 3,000 rpm after adding 0.5 ml trichloroacetic acid (10 g/100 ml). Then, 1 ml supernatant was combined with 0.2 ml ferric chloride (0.1 g/100 ml) and 1 ml distilled water. After letting the reaction for 10 min, the absorbance of the mixture was determined at 700 nm. The sample extract was not present in the blank control but it had all other reagents.

#### Statistical analysis

Each test was conducted at least three times, and IBM SPSS statistics version 21.0 was used to evaluate the results (IBM, Armonk, NY, USA). The significance level was defined at *P* < 0.05, and the pairwise multiple comparisons between treatments were made by performing Turkey’s HSD test after performing an analysis of variance (ANOVA).

## Results and discussion

### Purity and molecular weight of Qingke β-glucans

As presented in Table 1, the Qingke β-glucan content in all samples was above 85% and did not show any significant differences (*P* > 0.05), which indicated that the chemical components of QBG, QBG30, QBG60, and QBG90 were similar and the partial acid hydrolysis did not affect purity. The molecular weight of QBG was 510 (±18) KDa. Meanwhile, the molecular weights of QBG30, QBG60, and QBG90 were 280 (±18) KDa, 190 (±11) KDa, and 155 (±12) KDa, respectively. As the hydrolyzing time increased, the molecular weights decreased from 510 to 155 kDa. Additionally, the polydispersity index of QBG, QG30, QBG60, and QBG90 were shown to be 1.77 ± 0.03, 1.64 ± 0.04, 1.56 ± 0.05, and 1.69 ± 0.05, respectively.

**TABLE 1:** Molecular characteristics of Qingke β-glucan samples.

Sample	Hydrolysis time (min)	β–glucan content (%)	Mw (kDa)^a^	Mw/Mn^b^
QBG	0	82.17 ± 1.45	510 ± 18	1.77 ± 0.03
QBG30	30	84.07 ± 1.23	280 ± 15	1.64 ± 0.04
QBG60	60	86.16 ± 1.32	190 ± 11	1.56 ± 0.05
QBG90	90	87.83 ± 1.42	155 ± 12	1.69 ± 0.05

^a^Weight average molecular weight.

^b^Polydispersity index.

### Linkage analysis

The type and proportion of glycosidic linkages and other structural details on the Qingke β-glucan were determined by GC-MS (Table 2). The results showed that Qingke β-glucans mainly was composed of β-(1→3)-linked-D-glucopyranosyl and β-(1→4)-linked-D-glucopyranosyl, accounting for about 27 and 70% of the linkages, respectively. The ratio of β-(1→4)/β-(1→3) linkages of Qingke β-glucans ranged from 2.52 to 2.58. This indicated that the acid hydrolysis did not affect the chemical structures of Qingke β-glucans.

**TABLE 2:** Glycosidic linkage (mol%) of Qingke β-glucan samples.

Linkage	Linkage composition (mol%)			
	
	QBG	QBG30	QBG60	QBG90
(Glc*p*)1→	2.23	2.12	2.10	2.42
→3(Glc*p*)→1	27.66	27.42	27.32	27.76
→4(Glc*p*)→1	70.11	70.46	70.58	69.82
(1–4)/(1–3)	2.53	2.57	2.58	2.52

### Nuclear magnetic resonance analysis

**FIGURE 1:**
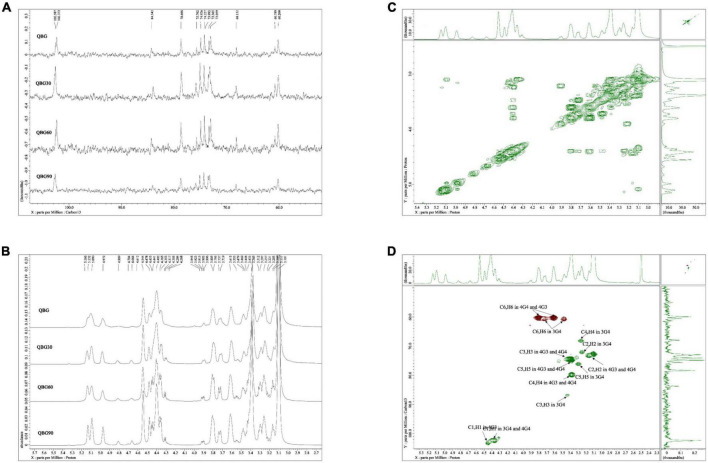
Nuclear magnetic resonance spectra: **(A)** ^13^C NMR spectrum of Qingke β-glucan samples, **(B)** ^1^H NMR spectrum of Qingke β-glucan samples, **(C)** ^1^H–^1^H COSY spectrum of QBG30, and **(D)** HSQC spectrum of QBG30.

The typical mixed-linkage (1→3) and (1→4)-β-glucan structure of the Qingke β-glucans was confirmed by the ^13^C NMR spectra (Figure 1A), which were comparable to oat and barley β-glucans (13). As shown in Table 3, the assignments represented that the C1 of the →4)-β-Glc*p*(1→3) (4G3) residue developed a resonance at 102.6 ppm because of the action of the (1→3)-β-linkage. The C1 group had a resonance at 102.4 ppm and was involved in forming (1→4)-β-linkage in →3)-β-Glc*p*(1→4) (3G4) and →4)-β-Glc*p*(1→4) (4G4) residues. Only one (1→3)-β-linkage was present, according to the C3 singlet at 84.3 ppm in the 3G4 residue. When compared to the 3-*O*-substituted residues, the resonances of C4 in 4-*O*-substituted residues moved to a lower field. At 60.7 and 60.3 ppm, the resonances for the C6 in the 3-*O*- and 4-*O*-substituted residues were found, respectively. The ratio of β-(1→4)/β-(1→3) linkages were computed by combining the anomeric signals of the 4-*O*-substituted and 3-*O*-substituted residues. The ratios of all β-glucans sample were between 2.52 and 2.58 and the anomeric carbons are similar for all Qingke β-glucans.

**TABLE 3:** Assignment of 13C NMR spectra of Qingke β-glucan samples.

Sugar residue	Chemical shift, ppm					
	
	C1	C2	C3	C4	C5	C6
→4)-β-Glcp(1→3)	102.6	73.4	74.2	78.7	74.9	60.3
→3)-β-Glcp(1→4)	102.4	73.0	84.3	68.2	75.7	60.7
→4)-β-Glcp(1→4)	102.4	73.0	74.3	78.7	74.9	60.3

Based on the ^1^H NMR spectra (Figure 1B), additional assignments of proton signals were analyzed according to the associated signals through the ^1^H–^1^H COSY spectrum of QBG30 (Figure 1C). The chemical shift range of the anomeric proton was determined and similar for QBG, QBG30, QBG60, and QBG90. We found that 4.45, 3.17, 3.72/3.47, 4.40, 3.26, 3.62/3.80, 4.36, and 3.1 ppm were H1 of 4G3, H2 of 4G3, H-6 of 4G3, H1 of 3G4, H1 of 3G4–2, H-6 of 3G4, H1 of 4G4, and H2 of 4G4, respectively. The correlations of H1/H2 and H2/H3 were observed, but 3.62 and 3.80 ppm were H6 of 4G4. The H3 of 4G3, H4 of 3G4, and H4 of 4G4 heavily overlapped. These results were consistent with those of previous studies (13).

The typical ^1^H–^13^C HSQC spectrum of the QBG30 (Figure 1D) exhibited two cross-peaks that 4.45, 4.40, and 4.40 ppm were H1 of 4G3, H1-3G4-2 and H1-4G4, which was correlated with carbons and protons. Despite having different molecular weights, the other samples (QBG, QBG60, and QBG90) had similar COSY spectrum and HSQC spectrum, which indicated that they had a similar structure.

### Fourier transforms infrared analysis

**FIGURE 2:**
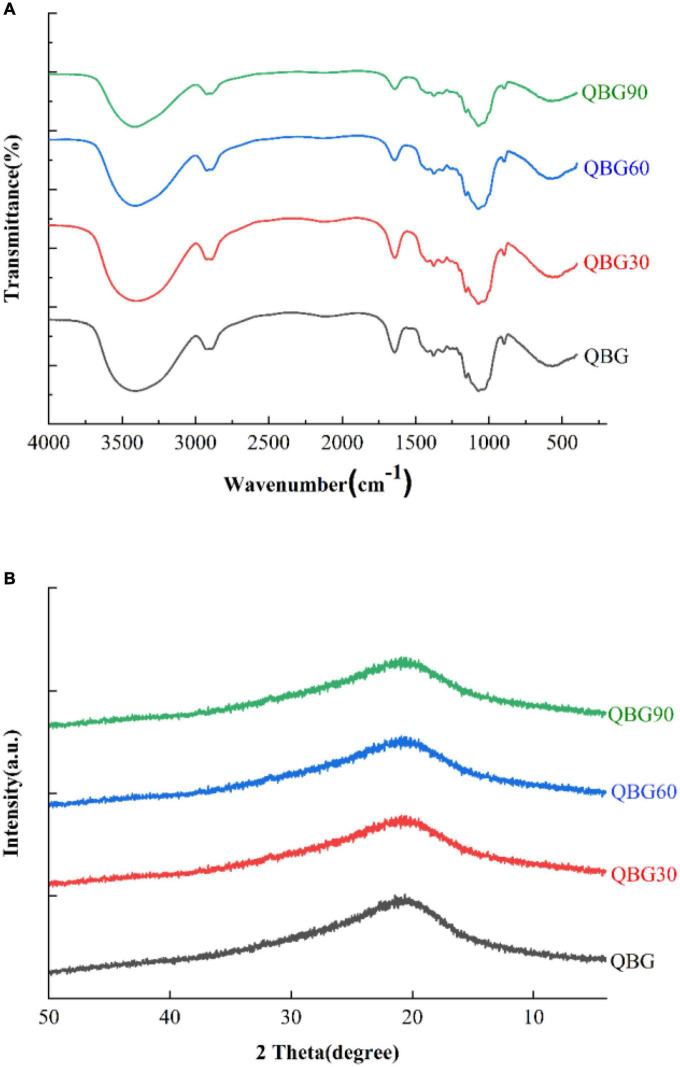
The FTIR spectra **(A)** and XRD spectra **(B)** of Qingke β-glucan samples.

The IR bands of all the β-glucans were comparable (Figure 2A). The bands at 3,416 cm^–1^ were attributed to typical vibrational modes of asymmetric and symmetric stretching of O-H groups that were present in β-glucans. Additionally, the intensity of the peak increased about 3,416 cm^–1^, which might have exposed more hydroxyl groups (16). The vibrational modes in the asymmetric and symmetric stretches of C-H groups may be related to the bands at 2,923 cm^–1^ (16). The bands at 1,640 cm^–1^ were associated with the deformation vibration absorption peak of a crystalline water hydroxyl group in Qingke β-glucan. At 1,155 and 1,071 cm^–1^, the β-glucans revealed two significant peaks, which might due to the difference in the number of β-(1→3) and β-(1→4) bonds that can cause C-O-C group vibrations (18). Additionally, the peak at around 897 cm^–1^ demonstrated the presence of β-linkages of polysaccharides. Bai et al. (16) identified the distinctive “anomeric region” (894 cm^–1^) as the β-linkage of pyranose of Qingke arabinoxylan. The intensity of the peak at 897 cm^–1^ increased, indicating that there was a greater chance of forming stronger hydrogen bonds. In summary, we found three broad and strong peaks at 3,416, 1,071, and 897 cm^–1^. The positions of the above-mentioned peaks were similar in all the β-glucan samples, indicating that acid hydrolysis could not influence the basic functional groups of Qingke β-glucan. The FT-IR spectral characteristics of β-glucan were similar with previously described cereal β-glucan (13).

### X-ray diffraction analysis

All samples had a similar XRD pattern (Figure 2B), indicating the presence of similar groups in Qingke β-glucan samples. The XRD pattern of Qingke β-glucan powder showed a broad diffraction peak and a typical high-molecular polymer diffraction pattern, revealing the presence of amorphous structure. The semicrystalline structure and characteristic peak of β-glucans at 2θ of 20.5 were similar to previous studies (16, 19). The large peak width indicated that the crystal forms were very similar. The peaks of the crystals of β-glucan were present in the crystalline part and lattice species, which suggested that they had macroscopic regularity and microscopic irregularity. The broad diffraction peaks of β-glucan implied that the spatial conformation of β-glucan had random coils. The large peak width indicated that the crystallization of β-glucan had macroscopic regularity and microscopic irregularity instead of a single or triple-helix structure.

### Analysis of the thermal properties

**FIGURE 3:**
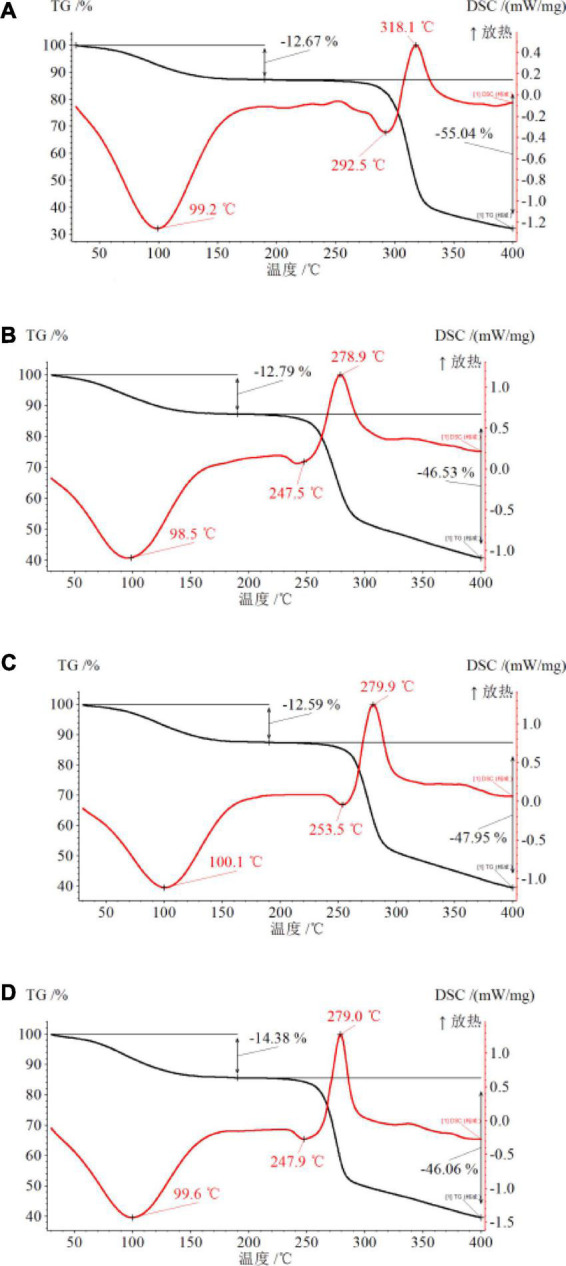
The DSC and TGA curves of QBG **(A)**, QBG30 **(B)**, QBG60 **(C)**, and QBG90 **(D)**.

Heating is a frequently used processing technique in the food processing industry. The heat processing denatures nutrients and the sensory quality because of the degradation of nutrients (20). Understanding the heat response of a polysaccharide is important for practical industrial applications. In this experiment, all β-glucan powders experienced the following similar two-stage heating process in an inert atmosphere. The first stage was the dehydration of physisorbed water molecules, and the second stage was the elimination and thermal degradation of surface-located functional groups of hydroxyl and oxygen. The effects of high-temperature treatment (30–400°C) on β-glucans were shown using DSC and TGA curves (Figure 3). Based on the TGA and DSC data, the β-glucans conjugate exhibited first-stage weight loss at 30–205°C, equivalent to the loss of moisture content by the destruction of inter-molecular and intra-molecular hydrogen bonds (21). At high temperatures (200°C), the chain structure of Qingke β-glucan changed, resulting in steady weight loss. Non-covalent bonds were broken, which was related to the large peak at 3,416 cm^–1^ (fourier transform infrared spectroscopy analysis) (16). The weight loss of QBG30, QBG60, and QBG90 conjugates in the first stage were similar to QBG. A larger mass loss of QBG is 55.04% at the second stage (206–360°C) (Figure 3A), with the mass loss of QBG30 (Figure 3B), QBG60 (Figure 3C), and QBG90 (Figure 3D) conjugated in the same stage was 46.53, 47.95, and 46.06%, respectively. At temperatures between 206 and 360°C, there was significant weight loss due to the degradation and carbonization of Qingke β-glucan (22). This might be due to the decomposition of β-glucan involved in the dissolution of chemical connections and breakdown of lengthy chains (23). Additionally, the peak temperature (Tp) of QBG was around 292.5°C while the Tp of QBG30, QBG60, and QBG90 were approximately 247, 253, and 247°C, respectively. Moreover, the Tp of DSC curves of QBG30, QBG60, and QBG90 shifted to lower temperatures, which indicated that the thermal stability of β-glucan increased with the raising molecular weight. When the temperature exceeded 360°C, the samples had lesser loss of mass and stabilized.

### Apparent viscosity of the Qingke β-glucan solution

**FIGURE 4:**
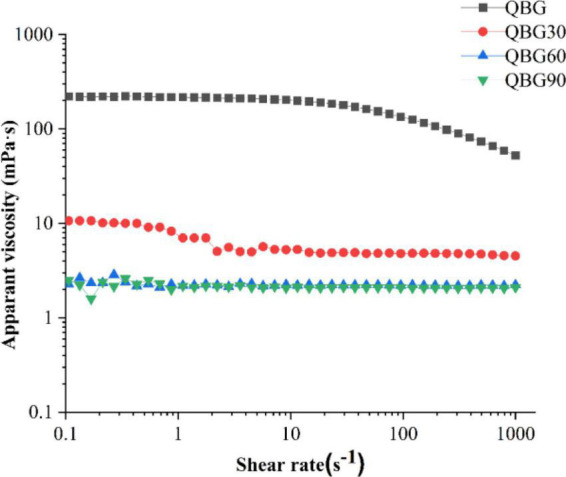
The apparent viscosity of β-glucan samples on shear rates at a concentration of 2%.

The changes in apparent viscosity of the Qingke β-glucan of different molecular weights were indicated in Figure 4. The viscosity of a 2% QBG solution displayed a second-order dependence on shear rate, with a Newtonian plateau at low shear rates and a shear thinning zone at higher shear rates, which was related to the normal behavior of a random coil (8). The behavior of QBG can be defined as pseudoplastic. The entangled structures constituted most of the aggregates in the 2% QBG solution, with tiny junction zones produced by succeeding cellotriosyl β-(1→3)-linked units. With increasing shear rate ranges, the β-glucans with low molecular weights (QBG30, QBG60, and QBG90) almost maintained their apparent viscosity. The viscosity of the Qingke β-glucan solution (QBG30, QBG60, and QBG90) remained constant with a Newtonian plateau. This was consistent with the findings of another study that the apparent viscosity of barley β-glucans and oat β-glucans showed Newtonian and pseudoplastic behavior (8).

The apparent viscosity of QBG was bigger than the viscosity of QBG30, QBG60, and QBG90. According to the findings of another study (24), the apparent viscosity increased with the increase of molecular weights. As the starch in QBG30, QBG60, and QBG90 was partly hydrolyzed during hydrolysis process, the other components (such as starch) of samples was another significant factor of apparent viscosity. The viscosity of the Qingke β-glucan (QBG, QBG30, QBG60, and QBG90) solution varied although their chemical compositions were similar. According to the same source, the variations in the viscosity of β-glucans might also be attributed to the differences in the molecular size, fine structure and other components, which affect the apparent viscosity.

### Antioxidant capacities of β-glucan

**FIGURE 5:**
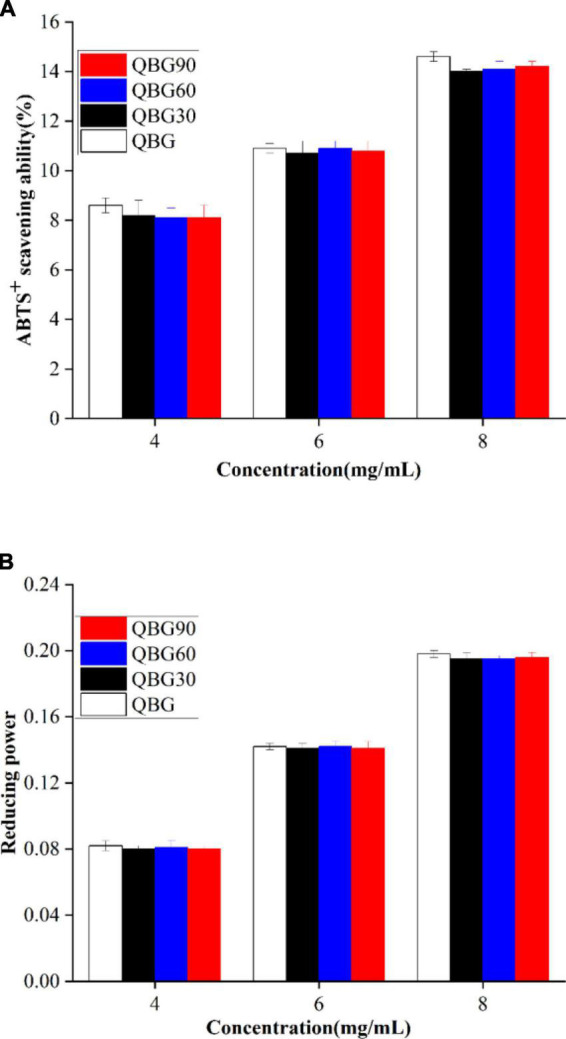
The ABTS radical cation scavenging ability **(A)** and reducing power **(B)** of the Qingke β-glucan samples.

The characteristics of phytochemicals, such as the molecular weight, glycosidic linking arrangement, different sources, and conformation, affect their antioxidant capacities (25, 26). The ability of β-glucans of different molecular weights to scavenge ABTS radical cations and their reducing power is shown in Figure 5.

ABTS^+^ free radicals were as an indicator to assess the antioxidant activity of plant polysaccharides. According to Shah et al. (18), the presence of many anomeric hydrogen atoms that are primarily obtained by the active free radicals can be responsible for the average ABTS radical cation scavenging ability of β-glucan. Since they act as antioxidants by donating a hydrogen atom and inhibit the chain reaction. The anomeric hydrogen atoms are commonly associated with the scavenging ability. The ABTS inhibition of Qingke β-glucan is shown in Figure 5A. The average ability of the β-glucans to scavenge ABTS radical cations of soluble β-glucans in Qingke showed a dose-dependent pattern. The β-glucans from *Dictyophora indusiata* showed a comparable concentration-dependent increase in the scavenging activity (27). The inhibition efficiency of Qingke β-glucans was in the range of 8.6–14.6% for QBG, 8.20–14% for QBG30, 8.10–14.1% for QBG60, and 8.1–14.2% for QBG90, respectively with the concentration from 4 to 8 mg/ml. Additionally, β-glucan samples of colored Qingke cultivars displayed a 7–20% scavenging effect on ABTS hydroxyl at identical concentrations (28), which were similar with our findings. This result suggested that Qingke β-glucans have hydroxyl radical scavenging ability, and the average ABTS radical cation scavenging capacities of Qingke β-glucan do not change considerably with molecular weights from 510 to 155 kDa. Previous studies showed that although the molecular weight decreased by roughly 1/20–1/50, the antioxidant activity was lowered only by about half (29). It showed that molecular weight and antioxidant activity are not synchronized. However, Bai et al. (16) showed that the inhibition rate on the ABTS radical of β-glucan decreased by 66.11–179.62% with an increase in the molecular weight, which was higher than the values in our study. The results might be attributed to the various species and molecular weights of β-glucan. Another reason might be that there was no change in the anomeric hydrogen atoms and anomeric carbon atom of β-glucans of acid hydrolysis. The FTIR and NMR results also showed that the β-glucan samples by acid hydrolysis did not cause changes in the anomeric hydrogen atoms and anomeric carbon atom.

Antioxidants function by donating hydrogen atoms to stop the chain reaction that produces free radicals, which prevents the development of peroxide. Since the reducing power and the antioxidant capacity are correlated. The reducing capacity can be used as a predictor of its prospective antioxidant capabilities. The reducing power of the β-glucans is shown in Figure 5B. The increase of absorbance also predicts an increase in the reducing power (30). The absorbance increased with the concentration of the β-glucan (from 4 to 8 mg/ml). The lowest absorbance was shown by QBG (0.082 ± 001) at 4 mg/ml, followed by QBG30 (0.08 ± 0.002), QBG60 (0.081 ± 0.001), and QBG90 (0.08 ± 0.002), and the highest absorbance was shown by QBG (0.198 ± 0.002) at 8 mg/ml followed by QBG30 (0.195 ± 0.003), QBG60 (0.195 ± 0.001), and QBG90 (0.196 ± 0.002). Khan et al. (30) demonstrated that the reducing activities of β-glucans extracted from *Agaricus bisporus* and *Pleurotus ostreatus* increased with the concentration from 1 to 5 mg/ml. The various molecular weights of β-glucans at the same concentration showed no significant variance in the reducing power. These results showed that the four Qingke β-glucans do not differ considerably as electron donors and the ability to stop free radical chain reactions by converting free radicals into more stable compounds.

## Conclusion

In this study, the QBG isolated from the hot water extract of Qingke by ethanol precipitation and ultrafiltration/centrifugation, and low-molecular-weight β-glucans (QBG30, QBG60, QBG60, and QBG90) produced by acid hydrolysis were investigated. The rheological behavior of β-glucan without and with acid hydrolysis can be described as pseudoplastic and Newtonian, respectively. The DSC curves of the β-glucans with high molecular weights showed the highest transition temperature. The antioxidant activities of Qingke β-glucans of different molecular weights were comparable because their functional groups did not differ from the results of FTIR and NMR. The findings of this study include details on the qualities and functions of Qingke β-glucan. In this study, we showed that Qingke β-glucan has nutraceutical potential that is significant for the development of functional foods and human health in general.

## Data availability statement

The original contributions presented in this study are included in the article/Supplementary material, further inquiries can be directed to the corresponding authors.

## Author contributions

LZ: investigation, data curation, methodology, formal analysis, roles, and writing—original draft. SL: conceptualization, formal analysis, validation, visualization, and writing—review and editing. JL: investigation, data curation, and methodology. JW: co-supervision, experimental design, and writing—review and editing. HC: supervision, conceptualization, funding acquisition, writing—review and editing, and project administration. All authors contributed to the article and approved the submitted version.
