# Supplementary material for: Effect of acid hydrolysis on the structural and antioxidant characteristics of β-glucan extracted from Qingke (Tibetan hulless barley)
Source: Front Nutr. 2022 Nov 10;9:1052901. doi: 10.3389/fnut.2022.1052901 (PMC9691401; doi:10.3389/fnut.2022.1052901)
Supplement: Supplementary file 1 [file Table_1.DOC]

**Table 1** Molecular characteristics of Qingke β-glucan samples

| Sample | Hydrolysis time (min) | β-glucan content (%) | Mw (kDa)^a^ | Mw/Mn^b^ |
| --- | --- | --- | --- | --- |
| QBG | 0 | 82.17±1.45 | 510±18 | 1.77±0.03 |
| QBG30 | 30 | 84.07±1.23 | 280±15 | 1.64±0.04 |
| QBG60 | 60 | 86.16±1.32 | 190±11 | 1.56±0.05 |
| QBG90 | 90 | 87.83±1.42 | 155±12 | 1.69±0.05 |

^a^ Weight average molecular weight.

^b^ Polydispersity index.

**Table 2** Glycosidic linkage (mol%) of Qingke β-glucan samples

| Linkage | Linkage composition (mol%) | | | |
| --- | --- | --- | --- | --- |
|  | QBG | QBG30 | QBG60 | QBG90 |
| (Glc*p*)1→ | 2.23 | 2.12 | 2.10 | 2.42 |
| →3(Glc*p*)→1 | 27.66 | 27.42 | 27.32 | 27.76 |
| →4(Glc*p*)→1 | 70.11 | 70.46 | 70.58 | 69.82 |
| (1–4)/(1–3) | 2.53 | 2.57 | 2.58 | 2.52 |

**Table 3** Assignment of 13C NMR spectra of Qingke β-glucan samples

| Sugar residue | Chemical shift, ppm | | | | | | |
| --- | --- | --- | --- | --- | --- | --- | --- |
|  | C1 | | C2 | C3 | C4 | C5 | C6 |
| →4)-β-Glcp(1→3) | 102.6 | 73.4 | | 74.2 | 78.7 | 74.9 | 60.3 |
| →3)-β-Glcp(1→4) | 102.4 | 73.0 | | 84.3 | 68.2 | 75.7 | 60.7 |
| →4)-β-Glcp(1→4) | 102.4 | 73.0 | | 74.3 | 78.7 | 74.9 | 60.3 |
